# Facile In Situ Photochemical Synthesis of Silver Nanoaggregates for Surface-Enhanced Raman Scattering Applications

**DOI:** 10.3390/nano10040685

**Published:** 2020-04-05

**Authors:** Zhen Yin, Huilin He, Zhenming Wang, Xiaoguo Fang, Chunxiang Xu, Dan Luo, Shouzhen Jiang, Yan Jun Liu

**Affiliations:** 1 Department of Electrical and Electronic Engineering, Southern University of Science and Technology, Shenzhen 518055, China; yinz3@sustech.edu.cn (Z.Y.); 11849548@mail.sustech.edu.cn (H.H.); 11510868@mail.sustech.edu.cn (Z.W.); 11849149@mail.sustech.edu.cn (X.F.); luod@sustech.edu.cn (D.L.); 2 State Key Laboratory of Bioelectronics, Southeast University, Nanjing 210096, China; xcxseu@seu.edu.cn; 3 Harbin Institute of Technology, Harbin 150001, China; 4 Shandong Provincial Key Laboratory of Optics and Photonic Device, School of Physics and Electronics, Shandong Normal University, Jinan 250014, China; jiang_sz@126.com

**Keywords:** photochemical synthesis, nanoaggregates, SERS, surface plasmon, hot spot, sensitivity

## Abstract

Recently, photochemical synthesis has attracted wide interest on in situ preparing the surface-enhanced Raman scattering (SERS) substrate with excellent performance, especially in a compact space and microfluidic channel. Herein, a facile, green and cost-effective approach to in situ photochemically synthesize silver nanoaggregates is demonstrated for SERS applications. By adjusting the photo-irradiation conditions, the morphologies and sizes of the silver nanoaggregates can be deliberately tailored. The synthesized silver nanoaggregates-based substrates exhibit a highly sensitive and reproducible SERS activity with a low detection limit of 10^−8^ M for 4-Aminothiophenol detection and relative standard deviation of 12.3%, paving an efficient and promising route for in situ SERS-based rapid detection in the environmental monitoring and food quality control.

## 1. Introduction

Nowadays, there is an ever-increasing need for high-performance analytical tools that can be applied in various fields ranging from life care, environmental monitoring, food safety to defense. Such analytical tools are generally required to be simple to make, low-cost, and easy to integrate. Along this line, surface-enhanced Raman spectroscopy (SERS) [1,2] seems to be a very promising tool due to its strong capabilities including label-free analysis, ultrahigh sensitivity, excellent selectivity, and multiplexing detection. SERS has been proven to be highly effective toward sensing of diverse analytes, e.g., metabolites, disease markers, pathogens, terrorism markers, hazardous pollutants, and illegal drugs for exploitation in biomedicine, food quality control, environmental monitoring, and security applications [3,4,5,6,7,8,9,10]. How the SERS substrate is fabricated and how well it performs will be the major determining factors that govern the final success of the SERS sensor. As is known, the enhancement effect of SERS is attributed to two major mechanisms, i.e., chemical and electromagnetic enhancement, which are supported by formation of charge transfer complexes and excitation of surface plasmons, respectively. Comparatively, electromagnetic enhancement factor can be several orders of magnitude higher than chemical one [11]. Therefore, plasmonic nanoparticles such as gold and silver nanoparticles (Au and Ag NPs) that possess strong localized surface plasmon resonance (LSPR) have been widely used as SERS substrates mainly due to their significantly LSPR-enhanced Raman signals [12,13,14].

In particular, silver nanomaterials have attracted widespread attention due to their excellent optical properties of wider LSPR adjustable range and sharper resonance band compared with other metallic materials. Usually, the surface morphology of silver nanomaterial plays an important role in the performance of SERS substrates by controlling particles size, shape [15,16], assembly and orientation distribution [17]. Various SERS-active substrates based on different shapes of nano-silver, such as silver nanocubes [18], nanowires [19], nanoprisms [20,21], nanoplates [22], nanobipyramids [23], nanopolyhedra [24], and nanodendrites [25,26], have been developed recently. Overall, there has always been a strong driving force behind the development of rapid, simple, and efficient methods for SERS substrates. From our perspective, silver nanoaggregates could be a more efficient SERS substrate due to plenty of hot spots from large quantities of nanoscale junctions and large surface areas. However, it is still a great challenge to achieve in situ and online synthesis of silver nanoaggregates via facile and cost-effective routes in a confined space and microfluidic channel for SERS applications.

Herein, we report a facile, green and cost-effective approach to produce silver nanoaggregates as effective SERS substrates, via a photochemical growth and deposition process. We have investigated the effect of photo-irradiation conditions on the morphology and size of the resultant silver nanoaggregates by scanning electron microscopy (SEM). The SERS performance of the silver nanoaggregates is evaluated using both Rhodamine 6G (R6G) and 4-Aminothiophenol (4-ATP) molecules. The synthesized silver nanoaggregate-based substrates exhibit a highly sensitive and reproducible SERS activity that is promising for in situ SERS-based rapid detection in the environmental monitoring.

## 2. Experimental

### 2.1. Photochemical Deposition

**Figure 1:**
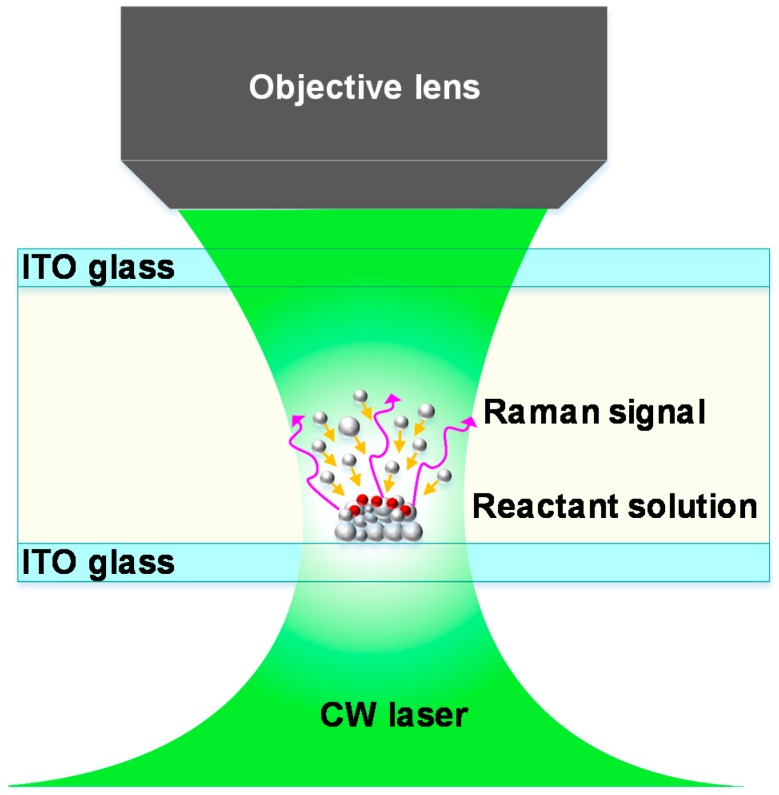
Schematic illustration of in situ photochemical deposition of silver nanoaggregates and SERS measurement. The gray and red spheres represent the silver atom and analyte molecule, respectively.

Photochemical synthesis was carried out inside a liquid cell, as schematically illustrated in Figure 1. The liquid cell was formed by assembling two pieces of cleaned indium-tin-oxide (ITO) glass substrates (size: 20 mm × 20 mm). The cell gap was controlled to be about 300 μm in thickness. Finally, the cell was filled with 43 μL of the reactant solution for the photochemical growth and deposition of silver nanoaggregates. The reactant solution was an aqueous mixture of silver nitrate (Xilong Scientific Co., Ltd., Shantou, China) and trisodium citrate (Sinopharm Chemical Reagent Co., Ltd., Shanghai, China) with equal concentrations of 5 × 10^−3^ M. The liquid cell was mounted on a motorized three-dimensional translation stage that was built in an optical microscope, and then irradiated with a focused laser (continuous wave laser, wavelength: 532 nm) via a microscope objective (20×, NA = 0.4).

The photochemical synthesis of the silver nanoaggregates can be described as the following [27,28]. The silver ions of Ag^+^ are firstly adsorbed on the ITO glass surface by electrostatic attraction. Upon laser irradiation, citrate is decomposed to produce electrons [29], and the silver atom is generated by addition of the electron to the silver ion by the following processes:

(1) $$\mathrm{Citrate}\overset{hv}{\to }\mathrm{acetone}-{1,3-\mathrm{dicarboxylate}+\mathrm{CO}}_{2}+e^{-}$$

(2) $${\mathrm{Ag}}^{+}+e^{-}\to {\mathrm{Ag}}_{0}$$

The reduced silver atoms will further aggregate to form a silver atom cluster. Combined with the negative electron of citrate, the silver atom cluster becomes the silver cluster of ${\mathrm{Ag}}_{n}^{-}$, which acts as the active sites for the reaction, and the surrounding silver ions are more easily reduced and deposited on the surface of the silver cluster [30]; thus, silver nanoaggregates are formed at the interface between the glass surface and reactive solution. The reactions below are included during this step.

(3) $${\mathrm{Ag}}_{n}+e^{-}\to {\mathrm{Ag}}_{n}^{-}$$

(4) $${\mathrm{Ag}}_{n}^{-}+{\mathrm{Ag}}^{+}\to {\mathrm{Ag}}_{n+1}$$

By adjusting the laser power and irradiation time, one can effectively control the morphology and size of the silver nanoaggregates that were further characterized by scanning electron microscope (SEM, Zeiss Merlin, Jena, Germany) with an acceleration voltage of 5 kV.

### 2.2. SERS Measurement

The SERS spectra were collected with a confocal Raman system (Ahpha300, WITec, Ulm, Germany). We used an objective lens (50×, Numerical aperture: 0.5) to focus the excitation laser light (working wavelength: 532 nm) onto the samples and collect the Raman signal to the spectrometer. Both R6G and 4-ATP molecules with different concentrations from 10^−4^ to 10^−8^ M were used to evaluate the SERS performance of the prepared substrate. The R6G and 4-ATP were dissolved in the deionized water and ethanol, respectively. The silver nanoaggregates were incubated in these probe molecular solutions overnight and then naturally dried for the SERS experiments.

## 3. Results and Discussion

**Figure 2:**
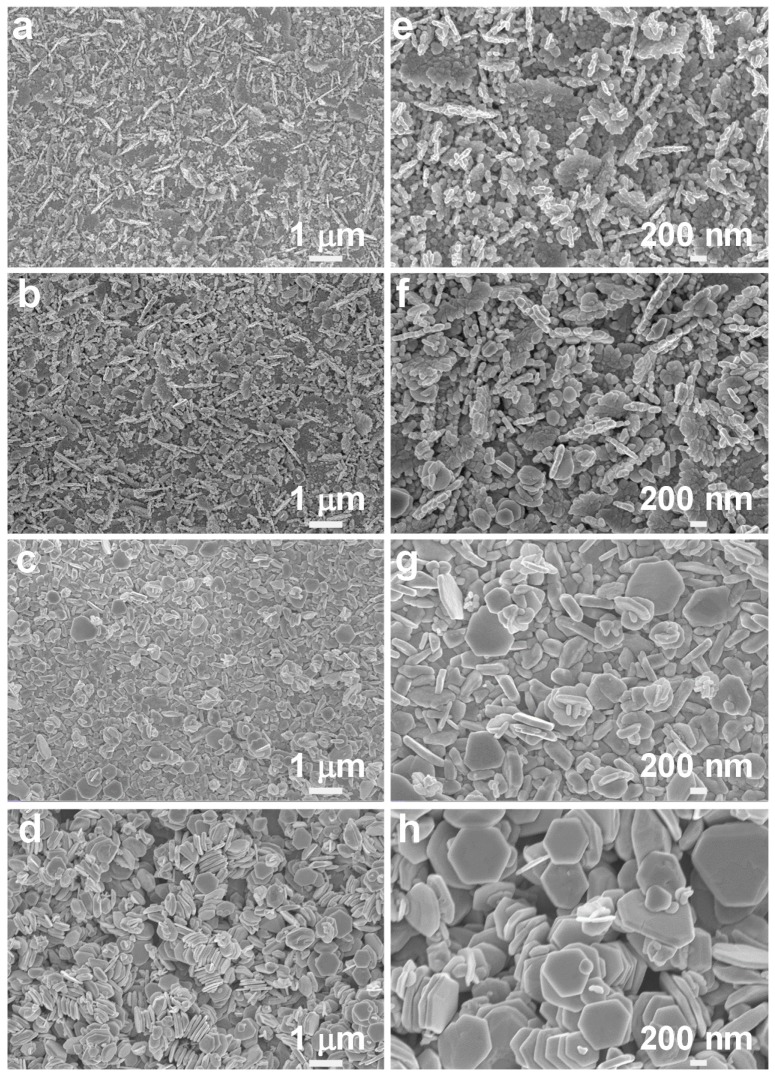
Low- and high-magnification SEM images of silver nanoaggregates prepared at irradiation intensities of (**a**,**e**) 1.5 × 10^5^ W/cm^2^, (**b**,**f**) 4.5 × 10^5^ W/cm^2^, (**c**,**g**) 8.0 × 10^5^ W/cm^2^, and (**d**,**h**) 1.2 × 10^6^ W/cm^2^, respectively, at a fixed irradiation time of 25 min.

It is well known that the SERS performance is highly dependent on the morphology and size of the silver nanoparticles. The control of irradiation intensity and time has a great effect on the morphology of the silver nanoaggregates substrate. Figure 2 shows a series of samples prepared under irradiation intensities of 1.5 × 10^5^ W/cm^2^, 4.5 × 10^5^ W/cm^2^, 8.0 × 10^5^ W/cm^2^ and 1.2 × 10^6^ W/cm^2^, respectively, with a fixed irradiation time of 25 min. From Figure 2a,e, we can see that at the relatively low irradiation intensity of 1.5 × 10^5^ W/cm^2^, silver nanoparticles are photochemically grown on the substrate with a particle size of 40–70 nm, and some silver nanoparticles aggregate vertically. As the laser intensity increases to 4.5 × 10^5^ W/cm^2^, the nominal size of silver nanoparticles increases, ranging from 50 to 100 nm, and more nanoparticles are agglomerated together, forming a large number of flaky nanoaggregates with sharp edges and corners, as shown in Figure 2b,f. Moreover, a small number of silver nanosheets with sizes of 150–200 nm appear. At the higher irradiation intensity of 8.0 × 10^5^ W/cm^2^, the shapes of most nanoparticles become flat nanosheets, and there are a few nanoparticles with truncated hexagons ranging from 200 to 450 nm, as shown in Figure 2c,g. From Figure 2d,h, at the highest irradiation intensity of 1.2 × 10^6^ W/cm^2^, almost all of the nanoparticles are interestingly transformed into hexagonally truncated nanosheets with the size of 500–700 nm. The formation of truncated hexagonal nanosheets is obviously related to the fast kinetics of the photochemical reaction caused by the high irradiation intensity. However, the detailed mechanism is still under investigation. Xue et al. found that the in-plane quadrupolar plasmon resonances is conducive to the growth of truncated hexagonal nanosheets by localizing energy on the edges and thus to promote silver deposition on the edges [31].

**Figure 3:**
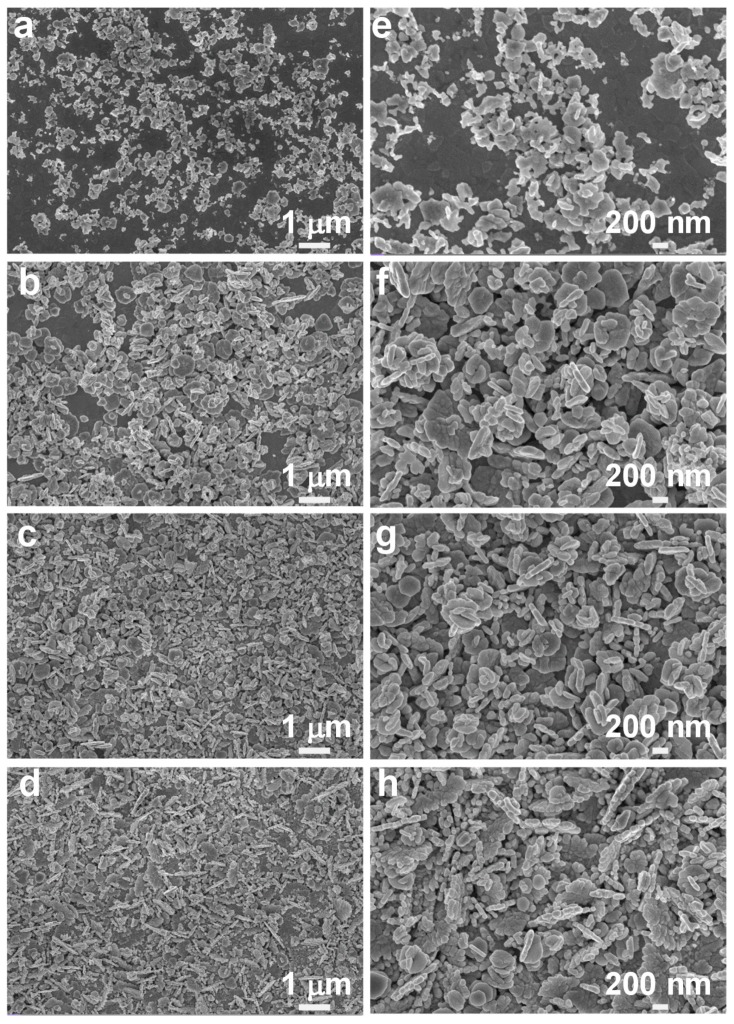
Low- and high-magnification SEM images of silver nanoaggregates prepared at the irradiation time of (**a**,**e**) 10, (**b**,**f**) 15, (**c**,**g**) 20, and (**d**,**h**) 25 min, respectively, at a fixed irradiation intensity of 4.5 × 10^5^ W/cm^2^.

Alternatively, one can also tune the morphology of the silver nanoaggregates by controlling the growth time of the silver nanoaggregates. Figure 3 shows the effect of irradiation time of 10, 15, 25, and 25 min, respectively, on the nanoaggregates’ morphology at the fixed irradiation intensity of 4.5 × 10^5^ W/cm^2^. As shown in Figure 3a,e, at the irradiation time of 10 min, there are fewer particles scattered on the ITO substrate, and most of the nanoparticles are agglomerated on the surface of the substrate. From Figure 3b–d and Figure 3f–h, as the irradiation time increases, the number of particles increases greatly, and more agglomerates with sharp edges and corners are formed. Meanwhile, numerous gaps between the nanoparticles are created both laterally and vertically. As a result, on one side, the silver nanoparticles themselves possess strong LSPR; moreover, strong plasmonic coupling also occurs between the neighboring nanoparticles/nanoaggregates. Hence, there are a great number of hot spots generated in the silver aggregates upon light excitation, which can enhance the localized electromagnetic fields directly [32,33,34]. On the other hand, both lateral and vertical nanogaps provide significant boost of the surface-to-volume ratio, which can further enhance the adsorption of the analyte molecules. These two effects could then act collectively and synergistically to enhance the SERS activity greatly.

**Figure 4:**
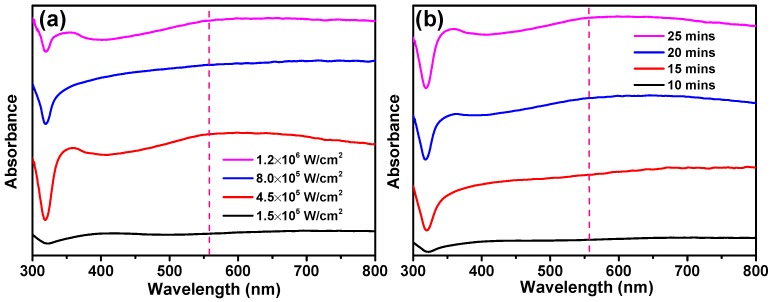
Absorbance spectra of the silver nanoaggregates-based SERS substrates prepared (**a**) at irradiation intensities of 1.5 × 10^5^, 4.5 × 10^5^, 8.0 × 10^5^, and 1.2 × 10^6^ W/cm^2^, respectively, with a fixed irradiation time of 25 min; (**b**) at irradiation times of 10, 15, 20, and 25 min, respectively, with a fixed irradiation intensity of 4.5 × 10^5^ W/cm^2^.

As is known, the SERS activity is highly dependent on the wavelength match between the LSPR resonance and excitation laser [35,36]. We therefore measured the absorbance spectra of the fabricated silver nanoaggregates-based SERS substrates, as shown in Figure 4. From Figure 4, we can see that a broad band appears within the visible range due to irregular shapes and various sizes of silver nanoparticles, and the strong LSPR coupling effect from the silver nanoaggregates. When the SERS substrate is fabricated with the irradiation intensity of 4.5 × 10^5^ W/cm^2^ and the irradiation time of 25 min, it has a characteristic absorption peak at ~560 nm, which is very close to the excitation laser wavelength (532 nm) used for SERS measurement (Figure 4a). From Figure 3 and Figure 4b, as the irradiation time increases, the number of silver nanoparticles increases greatly. As a result, more silver nanoaggregates and nanogaps are simultaneously formed, providing strong LSPR and plasmonic coupling.

**Figure 5:**
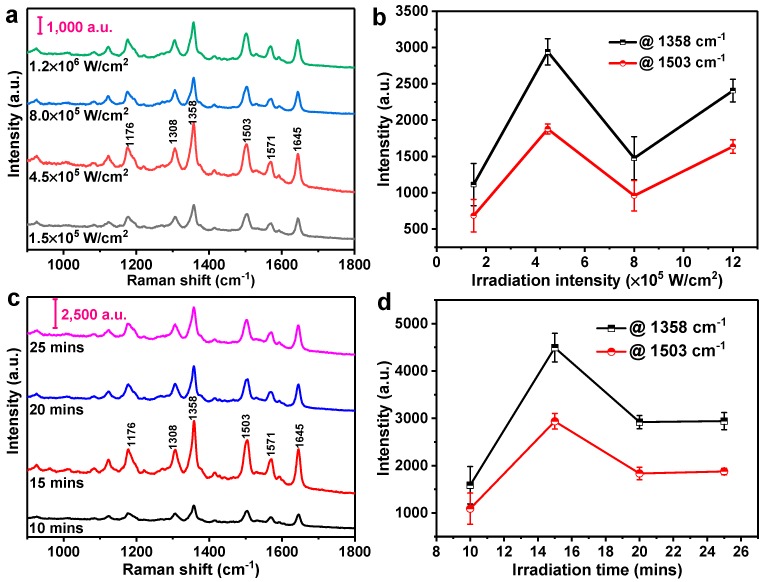
(**a**) SERS spectra of 10^−6^ M R6G adsorbed on the silver nanoaggregates prepared under different irradiation intensities at the fixed irradiation time of 25 min; (**b**) Raman signal intensities of R6G at 1358 cm^−1^ and 1503 cm^−1^ versus the irradiation intensities. (**c**) SERS spectra of 10^−6^ M R6G adsorbed on the silver nanoaggregates prepared under different irradiation time at the fixed irradiation intensity of 4.5 × 10^5^ W/cm^2^; (**d**) Raman signal intensities of R6G at 1358 cm^−1^ and 1503 cm^−1^ versus the irradiation time. The power of the excitation laser was 38.2 μw, and the acquisition time was 10 s for each spectrum.

The SERS activity of the synthesized silver nanoaggregates under different irradiation conditions was evaluated by using R6G as the probe molecule. From Figure 5a,c, the characteristic Raman peaks of 10^−6^ M R6G can be clearly observed for the silver nanoaggregates-based substrates that were prepared under different irradiation intensities and time. The characteristic Raman peaks of R6G at 1176, 1308, 1358, 1503, 1571, and 1645 cm^−1^ can be assigned to the in-plane vibration mode of the C–H bonds and the aromatic C–C stretching vibration mode, respectively. We can see that the intensity of these characteristic peaks highly depends on the irradiation conditions used to grow silver nanoaggregates. Figure 5b shows the intensities of Raman peaks at 1358 and 1503 cm^−1^, respectively, as a function of the irradiation intensities. The grown silver nanoaggregates at the irradiation intensity of 4.5 × 10^5^ W/cm^2^ show the highest intensities of both Raman peaks of R6G at 1358 and 1503 cm^−1^. The observed strong SERS signal can be attributed to the surface morphologies of the grown silver nanoaggregates. From the high-magnification SEM images (see Figure 2e–h), we can see that comparatively, there are a large number of nanoaggregates with sharper edges and corners in Figure 2f, providing much more hot spots to concentrate the electromagnetic field and hence resulting in much stronger SERS performance. Similarly, Figure 5d plots typical SERS intensity versus the irradiation time for the silver nanoaggregates in Figure 5c. With an irradiation time of 15 min, the highest SERS activity was obtained. From the high-magnification SEM images (see Figure 3e–h), there are many gaps between the nanoparticles that can effectively help adsorb more R6G molecules and subsequently improve the SERS performance. From Figure 5b,d, we can see that both the irradiation intensity and time have a significant effect on the SERS activity, which is essentially determined by the number of the created hot spots of the silver nanoaggregates. By varying the irradiation intensity and time and checking the corresponding SERS activity, we therefore achieved the optimal silver nanoaggregates-based substrate that was synthesized with the irradiation intensity and time of 4.5 × 10^5^ W/cm^2^ and 15 min, respectively. The achieved optimal SERS substrates were then used in our following experimental tests.

**Figure 6:**
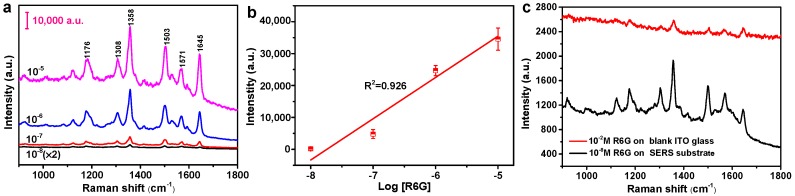
(**a**) SERS spectra of R6G with different concentrations on the silver nanoaggregates with the irradiation intensity and time of 4.5 × 10^5^ W/cm^2^ and 15 min, respectively. (**b**) Raman intensity of R6G at 1358 cm^−1^ as a function of different concentrations in log scale. (**c**) SERS signals of 10^−8^ M R6G on the silver nanoaggregates and 10^−2^ M R6G on the ITO glass substrate, respectively. The power of the excitation laser was 38.2 µW, and the acquisition time was 10 s for each spectrum.

With the optimal SERS substrates, we further investigated their sensitivity. Figure 6a shows the measured SERS spectra with different R6G concentrations, revealing that the limit of detection (LOD) for the R6G probe molecule is as low as 10^−8^ M. Figure 6b further plots the intensity of the typical SERS peak at 1358 cm^−1^ as a function of the concentration of R6G in log scale, which exhibits excellent linear dependence with a correlation coefficient (*R*^2^) of 0.926, hence allowing one to have quantitative analysis (for example, the concentration determination) of the analyte molecules. The excellent performance can be ascribed to the rich hot spots from the silver nanoaggregates. Figure 6c shows the SERS signal of 10^−8^ M R6G on the silver nanoaggregates-based substrate, and the Raman signal of 10^−2^ M R6G on ITO glass substrate separately. The enhancement distribution of SERS substrate is further quantified by the calculation of analytical enhancement factor (*AEF*) according to the equation given below [37]:

(5) $$AEF=\left(I_{SERS}\times C_{REF}\right)/\left(I_{REF}\times C_{SERS}\right)$$

where *I_SERS_* and *I_REF_* represent the peak intensity of the SERS signal and the normal Raman signal obtained from silver nanoaggregates as SERS-active substrate and ITO glass as reference substrate, respectively. *C_SERS_* and *C_REF_* are the concentrations of R6G solution deposited on the SERS and reference substrate, respectively. The peak intensity of 1358 cm^−1^ is chosen to calculate the *AEF*. Here, *I_SERS_* is 817 for 10^−8^ M R6G on the SERS substrate, and *I_REF_* is 159 for 10^−2^ M R6G deposited on the reference ITO glass substrate. Thus, the *AEF* is calculated to be 5.1 × 10^6^, indicating a high level of SERS performance. It is comparable to the SERS activity of silver colloids prepared by a γ-source radiolytic method [38].

**Figure 7:**
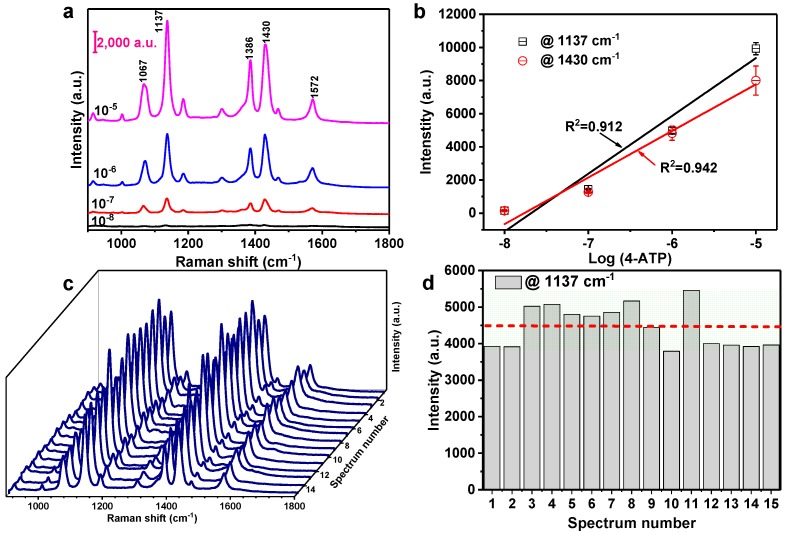
(**a**) SERS spectra of 4-ATP with different concentrations adsorbed on the same silver nanoaggregates-based substrate prepared under the irradiation intensity and time of 4.5 × 10^5^ W/cm^2^ and 15 min, respectively. (**b**) SERS intensity at 1137 and 1430 cm^−1^ for different concentrations of 4-ATP, the curves represent the linear fit to the experimental data. (**c**) Collected SERS signals of 4-ATP with the concentration of 10^−6^ M at 15 randomly selected positions. (**d**) Spot-to-spot intensity variation of the characteristic peak at 1137 cm^−1^ for the silver nanoaggregates-based substrate. The power of the excitation laser was 38.2 µW, and the acquisition time was 10 s for each spectrum.

To explore the feasibility of the silver nanoaggregate-based SERS substrate in practical applications, we have further investigated the detection of the other probe molecule, 4-ATP. The 4-ATP has been widely used as a chemical reagent, fine chemicals, pharmaceutical intermediates, and material intermediates. It can denature proteins in the human body once absorbed in the human body. Hence, the sensitive detection of trace 4-ATP molecules plays a significant role in human health. The SERS spectra of 4-ATP with the concentrations ranging from 10^−5^ to 10^−8^ M were collected on the prepared silver nanoaggregates. The dominating characteristic peaks of 4-ATP in the range from 900 to 1800 cm^−1^ are shown in Figure 7a. There are five distinct Raman peaks at 1067, 1137, 1386, 1430, and 1572 cm^−1^ of 4-ATP, which agree well with the previous report [39]. The relatively strong SERS peaks at 1137 and 1430 cm^−1^ were selected to characterize the relationship between the intensity of the SERS signals and the concentration of the 4-ATP in log scale. As indicated in Figure 7b, the linear fit for the Raman peaks at 1137 and 1430 cm^−1^ gives the correlation coefficient (*R*^2^) of 0.912 and 0.942, respectively, demonstrating the excellent capability for the quantitative analysis of 4-ATP.

In addition to the high detection sensitivity, the reproducibility of the SERS substrate is another critical factor for practical application. We carried out the collection of the SERS spectra from randomly selected positions within a circular irradiation area of 65 µm in diameter for evaluation. Figure 7c shows the measured SERS spectra of the 4-ATP molecules with the concentration of 10^−6^ M at 15 different positions on the same substrate. The obtained profiles of spectra are very similar with the selected position variation. There is neither a significant shift of the characteristic Raman peaks nor the obvious change of the peak strength, indicating the excellent reproducibility of the substrate. To analyze the relative standard deviation (RSD), the SERS sensitivity at 1137 cm^−1^ is quantified and plotted in the form of distribution histogram (see Figure 7d), while the horizontal dotted line reveals the average SERS intensity of 1137 cm^−1^ bands from 15 random positions and the signal fluctuation is shown in the shaded area. Moreover, the RSD of the peak intensity at 1137 cm^−1^ is 12.3%, further demonstrating the good reproducible property of the fabricated substrate. Therefore, the silver nanoaggregate-based SERS substrates with high sensitivity and excellent reproducibility could serve as a promising platform for the practical applications in the environmental monitoring and food quality control.

## 4. Conclusions

In summary, we have demonstrated a facile, green and cost-effective approach to synthesize silver nanoaggregates for SERS applications. The morphology and size of the silver nanoaggregates can be deliberately tailored by adjusting the irradiation conditions. Comparative experimental results on various substrates with different surface morphologies confirm that the interparticle spacing, sharp edges and corners are crucial for the SERS performance of silver nanoaggregates. With optimal irradiation conditions, the resultant silver nanoaggregates have a low LOD of 10^−8^ M for 4-ATP with the RSD of 12.3%, providing a promising platform for in situ SERS-based rapid detection in the environmental monitoring and food quality control.
